# COVID-19 vaccine hesitancy in six geopolitical zones in Nigeria: a cross-sectional survey

**DOI:** 10.11604/pamj.2022.42.179.34135

**Published:** 2022-07-06

**Authors:** Babatunde Oluwatosin Ogunbosi, Michael Abel Alao, Olayinka Rasheed Ibrahim, Adaeze Chikaodinaka Ayuk, Rasheedat Mobolaji Ibraheem, Chioma Laura Odimegwu, David Chibuike Ikwuka, Patricia Akintan, OreOluwa Morakinyo, Ayomide Toluwanimi Adeyemi, Ridwan Muhammad Jega, Temitayo Folorunso Olowookere, Olaseinde Emmanuel Bello, Bilkis Iyabo Owolabi, Abejegah Chukwuyem, Lawan Maryah Bukar, Aliu Rasaki, Amudalat Issa, Atana Uket Ewa, Regina Oladokun, Olusegun Olusina Akinyinka

**Affiliations:** 1 Department of Paediatrics, College of Medicine University of Ibadan and University College Hospital, Ibadan, Oyo State, Nigeria,; 2 University College Hospital, Ibadan, Oyo State, Nigeria,; 3 Department of Paediatrics, Federal Medical Centre, Kastina, Kastina State, Nigeria,; 4 Department of Paediatrics, College of Medicine University of Nigeria Enugu Campus, Enugu State, Nigeria,; 5 Department of Paediatrics and Child Health, University of Ilorin and University of Ilori, Kwara State, Nigeria,; 6 Department of Human Physiology, College of Health Sciences, Nnamdi Azikiwe University Nnewi Campus, Nnewi, Nigeria,; 7 Department of Paediatric, College of Medicine, University of Lagos, Lagos State, Nigeria,; 8 Department of Paediatrics, Usmanu Danfodiyo University Teaching Hospitals Sokoto, Sokoto State, Nigeria,; 9 Medical Department (Vaccines), GlaxoSmithKline Pharmaceutical Nigeria Ltd, Lagos, Nigeria,; 10 University of Medical Sciences Teaching Hospital, Akure, Ondo State, Nigeria,; 11 Department of Paediatrics, General Hospital, Ilorin, Kwara State, Nigeria,; 12 Infection Control and Research Centre, Community Health Department, Federal Medical Centre, Owo, Ondo State, Nigeria,; 13 Department of Paediatrics, University of Maiduguri Teaching Hospital, Maiduguri, Bauchi State, Nigeria,; 14 Department of Paediatrics, Gombe State University and Federal Teaching Hospital Gombe, Gombe State, Nigeria,; 15 Children Specialist Hospital Ilorin, Kwara State, Nigeria,; 16 Department of Paediatrics, University of Calabar Teaching Hospital, Calabar, Nigeria

**Keywords:** COVID-19, vaccine hesitancy, Nigeria

## Abstract

**Introduction:**

the high expectations that heralded the development of COVID-19 vaccines has been plagued with vaccine hesitancy (VH). The prevalence and associated factors of COVID-19 VH in the six geopolitical zones in Nigeria are explored.

**Methods:**

using a cross sectional survey, a pre-tested and validated questionnaire on a “Google form” was distributed via social media platforms and hard copies in the six geopolitical zones of Nigeria. Included, using a chain-reference sampling technique, were healthcare workers (HCW), university students and adults in the general population. Participants who expressed unwillingness to receive COVID-19 vaccine in the event of an available vaccine were considered to have vaccine hesitancy. Frequency and percentage were used to describe categorical variables. Multivariable logistic regression analysis was used to assess for factors associated with VH. Level of significance was set at 5% on two-sided tails test.

**Results:**

among 1615 respondents, mean (standard deviation) age was 36.7 (11.3) years, and 847 (52.4%) were males. More than half were healthcare workers (943; 58.4%), 97.4% had at least secondary level of education, and majority 60.5% belonged to the upper social class. The prevalence of VH was 68.5% (1107/1615), and 67.2% preferred foreign manufactured COVID-19 vaccines. On multivariable regression analysis, residence in Northeast (AOR 6.01, 95% CI 2.24, 16.10) and Northwest (AOR 3.33, 95% CI 1, 48, 7.48) geopolitical zones, the Igbo ethnic group (AOR 1.88, 95% 1.10, 3.22), Christians (AOR 1.86, 95% 1.10, 3.14), nurses (AOR 3.50, 95% CI 1.25, 9.80), pharmacist (AOR 5.82, 95% CI 2.12, 16.32) and participants without confidence in foreign vaccines (AOR 4.13, 95% CI 2.99, 5.72) were at higher likelihood of VH.

**Conclusion:**

vaccine hesitancy is high among adults in Nigeria, with higher likelihood among the Igbo ethnic group, Christian faith, residence in Northeast and Northwest geopolitical zones and those with an aversion to foreign-made vaccines. Targeted interventions are required for the desired COVID-19 vaccine uptake rate and herd immunity.

## Introduction

The world is in unprecedented times. The novel coronavirus disease which emerged in Wuhan, China in 2019 (COVID-19) continues to challenge every sphere of our lives and has negatively impacted the economies and health systems of almost every nation on the earth [1]. As at December 21, 2021 about 275 million cases were reported with about 5.4 million deaths [2]. Worse still, with almost two years of the pandemic, and counting, a cure remains elusive. Before now, non-pharmaceutical interventions were the main stay of prevention, while the world raced to develop a vaccine. Since the last quarter of 2020, various candidate vaccines emerged as part of the preventive options, though not without their challenges [3]. One of the most important challenges being vaccine hesitancy (VH) [4]. Vaccine hesitancy (VH) refers to delay or refusal in acceptance of vaccines despite availability of vaccine services [5]. The problem of VH has always been a public health challenge [4]. It is therefore not surprising that despite the unprecedented event that followed the COVID-19 outbreak, the humongous loss of life, and the long-term sequela consequent on this infection, the discovery of a vaccine for COVID-19 was met with resistance, suspicions, and hesitancy.

An effective vaccine implementation program involves a well-coordinated supply and distribution system, as well as effective uptake of the vaccines by the end-users. Vaccine hesitancy has, over the years, become a huge challenge to vaccine uptake by end-users [6]. Factors that contribute to VH are varied, complex and context specific, varying across time, place, and vaccines and are usually influenced by factors related to complacency, convenience and confidence [7,8]. In the United States (US), and Europe various VH groups often misuse scientific facts, and some misinformation to dissuade people from taking up vaccines [9]. The groups are often misinformed, and some deliberately misinform others. Nigeria has had her fair share of VH. The misinformation of polio vaccine being used as a form of birth control in the core north led to significant reduction in polio vaccine uptake in 2012 in the affected areas [10]. This led to setbacks in the polio eradication initiative with a resurgence of wild polio in two contiguous countries, and one non-contiguous country. It took the engagement of local religious, community and traditional leaders and extensive enlightenment campaign to reverse the trend [11-13].

The COVID-19 pandemic has come at a time of massive explosion of information due to the wide accessibility to the internet. Another important source of information in this unusual pandemic has been through social media. While this has helped to better understand and follow the trend of the pandemic globally, it has also brought on some challenges when inappropriate information is shared, especially through social media platforms. Recognizing this, the United Nations (UN) and the World Health Organization (WHO) aptly used the term “infodemic” [8]. The origin of the COVID-19 and attending circumstances also fueled distrust in many circles, some alluding to the possibility of it being a bioweapon developed in a laboratory experiment that went awry [8]. The advent of COVID-19, and the urgency required in development of an effective vaccine has seen the utilization of novel advanced technology like mRNA in the vaccine development process and shortened clinical trial phases [14]. These factors have raised concerns about the safety and potential of these vaccines to change human genetic makeup [15]. Consequently, the COVID-19 pandemic has been accompanied by an era of myths, suspicions, misinformation, and disinformation; all recipes for VH and tools for the proponents of VH.

Nigeria is the most populous country in Africa and 7^th^ in the world [16]. The COVID-19 pandemic in Nigeria is the 6^th^ largest in Africa in terms of cases and death of about 3,000 [17]. The country made efforts to access the COVID-19 vaccines for her populace, but considering her history and experience with VH, little is known about the potential magnitude of VH among the target populations and the factors that might be at play. Some studies have explored potential factors at play in VH in Nigeria. Not willing to pay for vaccines, concerns about vaccine safety and logistic challenges with accessing the vaccines have been reported as factors fueling VH [18,19]. Other factors include female gender, Christian religion, social class and non-clinical health workers [19-21]. More studies to better understand these factors are essential for the COVID-19 vaccine introduction and optimal uptake to achieve the desired results. Therefore, this survey sought to explore the prevalence of VH and the associated factors in the six geopolitical zones of Nigeria. This will contribute to information needed for a successful COVID-19 vaccination program. In addition, the findings from this study may guide policy, and interventions in the current fight against the COVID-19 pandemic in Nigeria.

## Methods

**Study design and duration:** this was a cross-sectional survey conducted between 1^st^ March 2021 and 30^th^ April 2021.

**Study setting:** this study was conducted across Nigeria's six geographical zones (Northwest, Northcentral, Northeast, Southwest, Southsouth and Southeast). To ensure a nationwide spread and support participant enrollment, health care providers from sixteen tertiary health centres in the 6 zones were the focal persons involved in the data collection process. Each of these geopolitical zones had one (1) tertiary level health facility research collaborator, except for the southwest, which had three (3) tertiary level health facility collaborators.

**Study population including the eligibility criteria:** included in the survey were individuals aged 18 years and above, resident in Nigeria, being COVID-19 vaccine naïve, frontline health workers, health sciences/non-health sciences student in a university or adult members of the general population.

**Sample size calculation:** the sample size required for this study was determined using the Raosoft sample size calculator for a single proportion with an estimated 50% prevalence of vaccine hesitancy (this assumption was adopted because as at the time of preparing this proposal there no available data on the prevalence of COVID-19 vaccine hesitancy in Nigeria). A minimum ample size of 377 has 80% power at an alpha level of 20% and probability of 0.05. A total of 1,805 participants were enrolled to allow for data accuracy and robustness.

**Sampling procedure:** participants in the study were sampled using a chain-reference sampling technique. The leaders of the groups and associations were contacted to introduce the study to colleagues and encourage their participation in the survey. Each respondent was asked to share the link with co-workers in the same geopolitical zone.

**Data collection:** a pre-tested and validated questionnaire on a Google form (Alphabet Incorporated, Mountain View, CA, USA) was used to collect data via the social media platforms. On the first page of the questionnaire, prior to the collection of data, consent was requested. Questionnaires was self-administered by study participants. However, in the Northwest, where internet access is limited and literacy levels are low, some consenting participants had the questionnaires administered by an interviewer.

**Description of the data collection tool and study variables:** questionnaire contained respondents´ demographic details of age, gender, marital status, level of occupation, occupation, geopolitical zone of residence at the time of survey, intention to collect COVID-19 vaccine or otherwise and preference as to locally or foreign manufactured COVID-19 vaccine. Vaccine hesitancy (VH) refers to delay or refusal in acceptance of vaccines despite availability of vaccine services [5]. For this study, participants who responded, “Definitely not” or “Probably not” to the question “If the COVID-19 vaccine is made freely available in Nigeria, would you take it? were categorized as having vaccine hesitancy,

**Data management and statistical analysis:** all submissions from participants were anonymized and handled in a strictly confidential manner. Those with incomplete data set were excluded from the data analysis. The data form submitted were analyzed using SPSS version 23 (IBM SPSS Statistics, Chicago, USA). Categorical data were described using frequencies and percentages, while continuous variables were presented using arithmetic mean and standard deviation. Univariate logistic analysis was performed to identify crude odds for VH which was adjusted for confounders with multivariable logistic regression analysis. Level of significance was set at 5% on two-sided tails test.

**Ethical consideration:** ethical approval (OYSERB AD13/479/44121B) was obtained from the Ministry of Health Ethics Committee of Oyo State where the lead researcher resides. Consent statement was included in the first page of the survey tool and participants would only proceed if consent was given. There was no participant identifier in the survey tool and all other sources of potential patient identifier were anonymised before data was analysed. The study was conducted in accordance with the Helsinki declaration.

## Results

**Figure 1:**
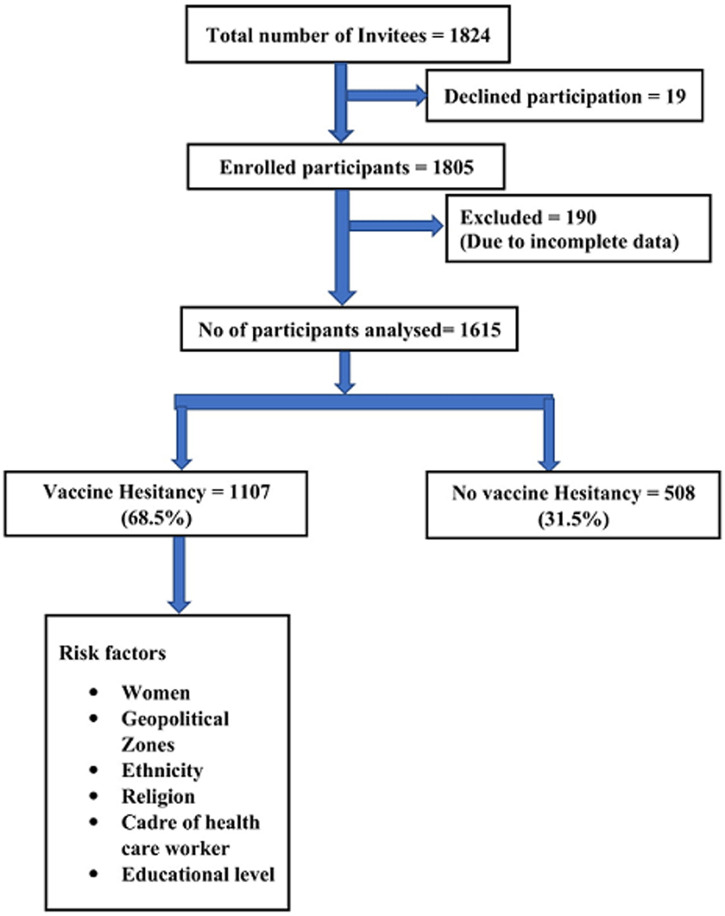
flow chart of the recruited study participants and prevalence of vaccine hesitancy

**Demographic characteristics of study population:** of the 1824 invited to take part in the study, 19 declined and thus 1,805 were enrolled of which 190 were excluded because of incomplete data, and data from 1615 respondents were analyzed (Figure 1). The mean (standard deviation) age of the participants was 36.7 (11.3) years, and most were males, 847 (52.4%). More than half (977; 60.5%) of the study participants were in the upper social class. Most of the participants761 (44.9%) were of the Yoruba ethnic group, followed by Igbo 409 (25.3.0%). The highest number of the respondents were from the southwest geopolitical zone (495; 30.7%) while the northeast (49; 3.0%) had the least number of respondents. Most of the respondents had at least secondary level of education (97.4%). More than half of the study participants were health care workers (943; 58.4%). Of the health care workers, medical doctors constituted the largest number of respondents (623; 66.1%). Further details are shown in Table 1.

**Table 1:** demographic characteristics of study population

Variables	Number (N) 1615	Percentage (%)
**Age group (years)**		
18 - 25	364	22.5
26 - 35	455	28.2
36 - 45	467	28.9
> 45	329	20.4
**Gender**		
Female	768	47.6
Male	847	52.4
**Tribe**		
Hausa	95	5.9
Igbo	409	25.3
Others	350	21.7
Yoruba	761	47.1
**Location**		
North-Central	472	29.2
North-East	49	3.0
North-West	123	7.6
South-East	328	20.3
South-South	149	9.2
South-West	494	30.7
**Occupation**		
Senior public servants, professionals, managers, large-scale traders, contractors	1014	62.8
Intermediate grade public servants, senior school teacher	125	7.7
Junior school teachers, drivers and artisans	31	1.9
Petty traders, messengers, labourers, and similar grades	47	2.9
Unemployed, full-time housewives, students, and farmers	398	24.7
**Educational level**		
Postgraduate	784	48.6
Tertiary	689	42.6
Primary/secondary	142	8.8
**Social class**		
Upper	977	60.5
Middle	496	30.7
Lower	142	8.8
**Religion**		
Muslims	1108	68.6
Christians	483	29.9
Others	24	1.5
**Study group N=1615**		
General adult population	255	15.8
Healthcare worker	943	58.4
Student	417	25.8
**Health care workers n=943**		
Doctor	623	66.1
Lab scientist	39	4.1
Nurse	76	8.1
Others	123	13.0
Pharmacist	82	8.7
**Students n=417**		
Health sciences	303	72.7
Non-health sciences	114	27.3

# Includes no formal education

**Intention to receive COVID-19 vaccination:** of the 1615 respondents, 1107 were not willing to accept COVID-19 vaccine giving a vaccine hesitancy prevalence of 68.5%. A higher percentage of respondents 67.2% (1086/1615) had confidence in foreign manufactured COVID-19 vaccines, compared to 52.8% (853/1615) with confidence in locally manufactured vaccines.

**Figure 2:**
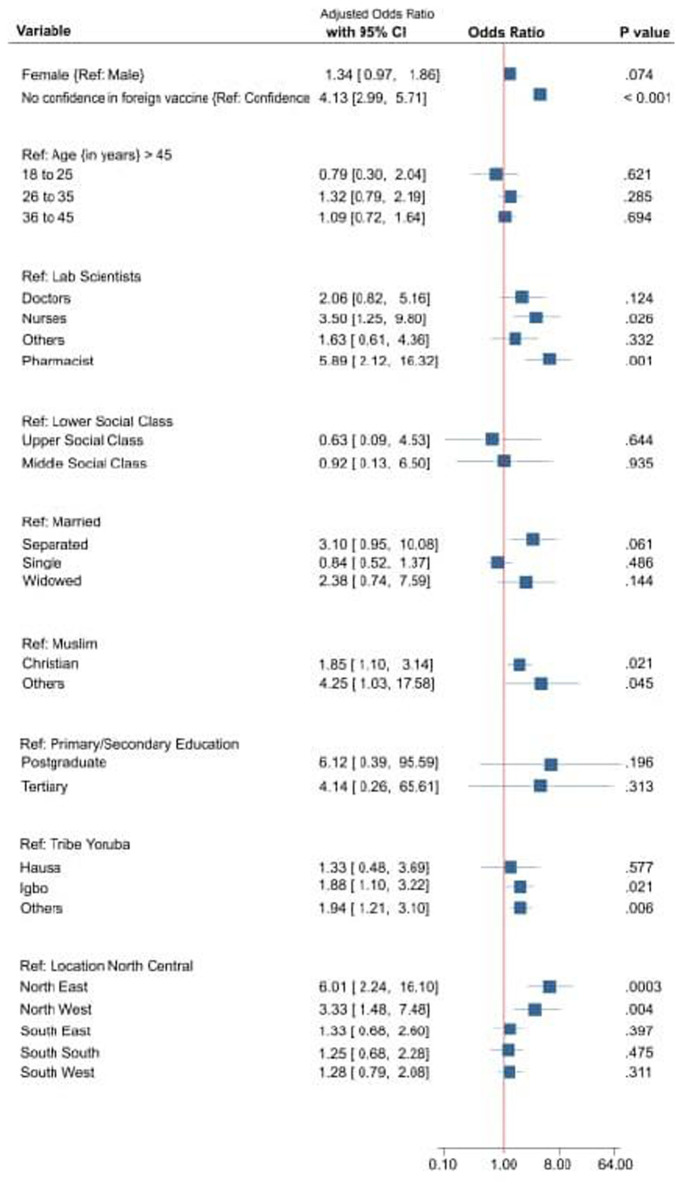
factors associated with vaccine hesitancy on multivariable regression analysis

**Socio-demographics factors associated with vaccine hesitancy:** on univariate analysis, of the eleven social demographic characteristics observed in this study, only age and marital status were not statistically significantly associated with vaccine hesitancy Table 2. On multivariable analysis, age was not significantly associated with VH. The women more than men had higher crude OR (Odds Ratio) 1.371 (95% CI: 1.11, 1.69) for VH. Among the study groups, health sciences students more than health works and non-health science students had a higher unadjusted odd 1.96 (95% CI; 1.192, 3.21, P= 0.008) other details are shown on Table 2. Adjusting for confounder on logistic regression residence in Northeast (AOR 6.01, 95% CI 2.24, 16.10) and Northwest (AOR 3.33, 95% CI 1, 48, 7.48) geopolitical zones compared with Northcentral had higher odds of VH. Also associated with higher likelihood of VH were belonging to Igbo ethnic group (AOR 1.88, 95% 1.10, 3.22), and the Christian faith (AOR 1.86, 95% 1.10, 3.14). Similarly, nurses (AOR 3.50, 95% CI 1.25, 9.80) and pharmacist (AOR 5.82, 95% CI 2.12, 16.32) were at higher likelihood of VH. Respondents without confidence in foreign manufactured COVID-19 vaccines, compared with those with confidence, were four times more likely to have VH. The details of adjusted odds for other factors associated with VH are shown graphically on the forest plot in Figure 2.

**Table 2:** socio-demographics associated with COVID-19 vaccine hesitancy amongst the respondents on univariate analysis

Variables	Categories	Vaccine hesitancy		Unadjusted OR (95% CI)	P-value
		No	Yes		
**Age (years**	> 45 (Ref)	236 (71.7)	93 (28.3)		
	18-25	239 (65.7)	125 (34.3)	1.327 (0.961, 1.833)	0.086
	26-35	316 (69.5)	139 (30.5)	1.116 (0.817, 1.525)	0.490
	36-45	314 (67.2)	153 (32.8)	1.236 (0.909, 1.683)	0.177
**Gender**	Male (Ref)	607 (71.7)	240 (28.3)		
	Female	498 (64.8)	270 (36.2)	1.371 (1.111, 1.692)	0.003
**Study group**	Non-health science students (Ref)	88 (72.2)	26 (22.8)		
	HCWs	672 (71.3)	271 (28.7)	1.365 (0.862, 2.161)	0.185
	Health science students	192 (63.4)	111 (36.6)	1.957 (1.192, 3.213)	0.008
	Gen. adults	153 (60.0)	102 (40.0)	2.256 (1.363, 3.736)	0.002
**Education**	#Primary/secondary (Ref)	111 (78.2)	31 (21.8)		
	Tertiary	461 (66.9)	228 (33.1)	1.771 (1.154, 2.719)	0.009
	Postgrad.	533 (68.0)	251 (32.0)	1.682 (1.102, 2.580)	0.016
**SEC**	Lower (Ref)	115 (81.0)	27 (19.0)		
	Middle	690 (70.6)	287 (29.4)	2.783 (1.763, 4.391)	< 0.001
	Upper	300 (60.5)	196 (39.5)	1.772 (1.140, 2.754)	0.011
**Study sites**	Northcentral (Ref)	376 (79.7)	96 (20.3)		
	Northwest	27 (55.1)	22 (44.9)	1.983 (1.280, 3.070)	0.002
	Northeast	81 (66.4)	41 (33.6)	3.191 (1.741, 5.849)	<0.001
	South	168 (51.2)	160 (48.8)	2.496 (1.676, 3.718)	<0.001
	Southeast	91 (61.1)	58 (38.9)	3.730 (2.731, 5.094)	<0.001
	Southwest	362 (73.1)	133 (26.9)	1.439 (1.067, 1.942)	0.017
**HCWs**	Laboratory scientist (Ref)	31 (79.5)	8 (20.5)		
	Pharmacists	48 (58.5)	34 (41.5)	2.745 (1.124, 6.703)	0.027
	Doctors	452 (72.6)	171 (27.4)	1.466 (0.661, 3.253)	0.347
	Nurses	49 (64.5)	27 (35.5)	2.135 (0.861, 5.295)	0.102
	Porters and maids	432(64.3)	240(35.7)	2.153 (0.974, 4.758)	0.058
	Others	93 (75.6)	30 (24.4)	1.250 (0.519, 3.012)	0.619
**Tribe**	Yoruba (Ref)	597 (78.4)	164 (21.6)		
	Hausa	62 (65.3)	33 (34.7)	1.938 (1.228, 3.058)	0.004
	Igbo	222 (54.3)	187 (45.7)	3.066 (2.364, 3.978)	<0.001
	Others	224 (64.0)	128 (36.0)	2.048(1.550, 2.705)	<0.001
**Marital status**	Married (Ref)	661 (69.5)	290 (30.5)		
	Widow(er)	10 (55.6)	8 (44.4)	1.823(0.712, 4.667)	0.210
	Separated	15 (60.0)	10 (40.0)	1.520 (0.675, 3.423)	0.313
	Single	419 (67.5)	202 (32.5)	1.099 (0.884, 1.366)	0.395
**Religion**	Muslims (Ref)	383 (79.3)	100 (20.7)		
	Christians	706 (63.7)	402 (36.3)	2.181 (1.695, 2.805)	<0.001
	Others	16 (66.7)	8 (33.3)	1.915 (0.797, 4.602)	0.146
**Confidence in foreign manufactured vaccine**	Complete confidence (Ref)	822 (75.8)	252 (24.2)		
	No confidence	283 (53.3)	248 (46.7)	2.749 (2.206, 3.426)	<0.001

OR -odds ratio; CI-Confidence Interval; SEC- Social economic class. #Included non-formal education

## Discussion

This study sought to evaluate prevalence of vaccine hesitancy among health care workers, university students and general adult population, and associated factors in the six geopolitical zones of Nigeria. A higher likelihood of VH was found in the Northwest and northeast geopolitical zones of Nigeria, among nurses, pharmacists, Christians, and those of Igbo or other minority tribes in Nigeria. The prevalence of vaccine hesitancy in this study was high and compared with most studies from Nigeria and Africa [19-24]. In contrast, rather lower prevalence of VH have been reported from the USA, China, Italy, United Kingdom and other Asian countries [25-31]. Reasons advanced for the observed VH include safety concerns, rapidity with which the vaccines were developed and general mistrust of government led health interventions [25].

The higher odds of VH in the north, especially in the Northeast and Northwest geopolitical zones, may be due to their traditional homogeneity and previously experienced hesitancy to immunisation programmes, most notably the polio eradication campaign [12,13,32]. Also, these geopolitical zones of the country are known to have lower coverage rates of routine childhood immunisation compared to other parts of the country. These may be indicative of the general low uptake of orthodox health care services of persons in these geopolitical zones. It is therefore imperative that traditional and religious leaders in these zones be engaged to facilitate uptake of the COVID-19 vaccine in these areas as has been done in the past with routine childhood immunisation and particularly during rejection of polio eradication vaccination campaigns [13,32].

As with other studies in Nigeria, a high likelihood of VH has been observed among health care workers [20] and in this study, nurses and pharmacists were found to have a significantly higher likelihood of VH than the general adult population. This contrasts with observations from the United States of America, France and Saudi Arabia [29,33,34]. The observed contrast may be because health care workers in these countries are used to routine vaccination against seasonal flu and other work-related health hazards. In line with global best practice, most COVID-19 vaccine roll-out programmes, including those in Nigeria, have prioritized HCW because they are a high-risk group. In the aftermath of the COVID-19 pandemic, and subsequent waves of the pandemic, there have been reports of nosocomial transmission and death among HCW [35,36]. This has also been linked to HCW deaths, and a decrease in available workforce to deal with the strains on the health system caused by the pandemic [35]. A significant increase in COVID-19 vaccine uptake among HCW is therefore critical. Furthermore, health care workers are regarded as reliable sources of health information, particularly for vaccine programmes in developing countries [37]. The COVID-19 pandemic has been bombarded with misinformation from a variety of sources, some of which have been proven to be false. Situations in which HCW do not receive the vaccine may affect the efforts to protect this vulnerable group, and have a negative impact on their ability to contribute to the positive messaging to reinforce the importance of receiving COVID-19 vaccine, and this may potentially lower vaccine uptake for the general population [38]. Also, HCWs not receiving vaccine may contribute to the dissemination of the virus to the patients.

Other demographics associated with higher likelihood of COVID-19 VH, as observed in other studies in Nigeria, include being of the Christian faith [19,21]. The effect of beliefs, whether religious or cultural, on VH is well-documented. These have been shown to influence individual or community perceptions of confidence or fuel complacency, both of which are important constructs in determining vaccine hesitancy [39]. When belief systems suggest that an individual is not at risk from a specific disease, it gives them the confidence that they are not at risk from such infection and thus fuels hesitancy in accepting vaccines that protect against the disease. Similarly, as in COVID-19, where a definite cure is not yet available, the Christian community's belief in a supernatural power to heal may contribute to the higher likelihood of VH observed among this group. The novel technologies and innovations used in the development of the COVID-19 vaccine were, in some religious parlance, indeed linked with the mark of the beast associated with the end time in the Christian faith. This, among other things, could be contributing to the findings in this study. This supports the need for engagement with religious and socio-cultural groups to increase COVID-19 uptake. Such approaches were used in the launch of the COVID-19 campaign in Nigeria, with the role of vaccines spearheaded by political, religious, and community leaders, and in some cases broadcast on electronic media platforms to encourage greater participation and acceptance.

Apart from the Hausa ethnic group, the Igbos and other Nigerian ethnic groups had higher likelihood of VH. Our findings support report of poor vaccine uptake among these subpopulations reported in Northern Nigeria and in the Eastern part of Nigeria [40]. Efforts should be concentrated on this vulnerable population. These subpopulation's clerics, traditional leaders, opinion leaders, and political commentators must be invited to a round table discussion. In order to achieve the desired hard immunity, they should be involved as advocates for vaccine uptake. The COVID-19 pandemic has been attended with lots of suspicions as to the origin and interventions to stem its tide [41]. While some suggests the pandemic was a result of laboratory experiment that went awry, others have alluded to the potential of the vaccines to negatively affect our genetic makeup. Indeed, early reports of blood clots and some deaths from early recipients of some type of COVID-19 vaccine has helped to fuel some of these narratives [42]. Not surprising, in this study, respondents who had no confidence in foreign manufactured vaccines were four times more likely to have VH, compared to their counterparts with confidence in these vaccines. Therefore, interventions to build public trust in the COVID-19 vaccine have been recommended to overcome some of these barriers to improving uptake of the COVID-19 vaccine [41]. These interventions must attend to peculiarities in each locale and approached with utmost sincerity.

Gender did not appear to influence the likelihood of VH in this study. There has been contrasting findings on the role of gender on VH from previous studies. While some suggest an influence, others have not found an association [21,29,33,34]. Gender may play a role in risk perception and experiences with health services which could in turn affect VH. Males are known to generally have poor health seeking behaviour than the women folk. Also, women could display higher likelihood of VH because they are frequently exposed to immunisation programmes and have seen their wards have adverse events following immunisation [43-45]. In traditional African societies, the female counterpart is usually exposed to adult vaccinations during antenatal clinics, and is frequently tasked with transporting infants to clinics for their immunisation series [46,47]. Their prior negative experience with adverse events following immunisation, as well as encounters with unfriendly health workers, may negatively influence VH. At yet it appears that the role of gender in COIVD-19 VH remains unclear.

Vaccine hesitancy was not significantly associated with social classes and student population on regression analysis. In terms of public health issues, student populations have been described as highly understandable and participatory groups. Prior to and during the COVID-19 pandemic, studies found that health sciences students had lower rates of VH due to their higher health literacy and contact with health care settings [27,48,49]. In contrast with other studies in both developing and developed countries, a higher VH rate was associated with the lower socioeconomic status and level of education in this study. The underrepresentation of the lower social class in the survey, despite concerted efforts to ensure equitable enrolment, may be the cause of the observation in the current study. It appears that varying context will continue to determine attitudes towards the COVID-19 vaccine and associated VH. This call for an on-going revision and studies on the subject matter as the response to the COVID-19 is sustained in the face of different waves and mutants COVID-19 strains experienced thus far.

While this study is unique in the light of its large size and it is also one of the few to have examined VH across Nigeria's six geopolitical zones, its generalizability across the country may still be limited by lack of external validity. It therefore calls for in-depth studies of specific homogenous populations in different parts of the country to examine the different constructs that may affect VH in the specific population. Other limitations include the method of data collection method. Most of the participants were contacted via electronic platforms, which may have excluded individuals who are not on those platforms or are not served by those platforms. It also, by reason of the electronic data deployed, may have excluded individuals without access to such electronic tools. However, considering the uniqueness and anticipated challenge of this in the Northwest geopolitical zone, some participants were enrolled using an interviewer-administered paper version of the data collection tool. In addition, though measures were taken to ensure enrolment of participants from specific geopolitical zones and professional groups as earlier stated, utilizing social media platforms cannot guaranty this was so in all cases. Furthermore, the timing of this study coincided with the introduction of the COVID-19 vaccine in Nigeria, and the sensitization campaign may have influenced individual vaccine acceptance decisions. The impact of this could also vary depending on the effectiveness of messaging and strategy deployed in different geopolitical zones. Lastly, while this study contributes to literature and further understanding of COVID-19 VH, it however still has other limitations like residual confounding as not all possible factors that may influence VH has been explored, lack of generalizability has been mentioned above and causality of associated factors discussed earlier is not established.

## Conclusion

This study has highlighted the diverse levels of COVID-19 vaccine hesitancy among a range of socio-demographic spectra in Nigeria. It contributes to other reports on COVID-19 vaccine hesitancy in Nigeria and adds some perspectives to possible interventions to mitigate this challenge. There is a need for targeted messaging on COVID-19 vaccination for improve uptake across the country.

### What is known about this topic

- Uptake of COVID-19 vaccine has been relatively low in most low- and middle-income countries compared to the developed countries;
- Vaccine hesitancy has been identified as obstacles to vaccine uptake with other vaccine preventable diseases in Nigeria; the magnitude of COIVD-19 vaccine hesitancy and the factors at play in Nigeria remains unclear.

### What this study adds

- The COIVD-19 vaccine hesitancy is high across the 6 geopolitical zones in Nigeria;
- It has also shown the disparity across the zones, with a higher likelihood of vaccine hesitancy in the Northern parts of the country, those of the Christian faith, Igbo ethnic group, nurses, pharmacists and among those without confidence in foreign manufactured COVID-19 vaccines.
